# Tissue Doppler Imaging in Coronary Artery Diseases and Heart Failure

**DOI:** 10.2174/157340312801215755

**Published:** 2012-02

**Authors:** Michele Correale, Antonio Totaro, Riccardo Ieva, Armando Ferraretti, Francesco Musaico, Matteo Di Biase

**Affiliations:** University of Foggia, Department of Cardiology

**Keywords:** Imaging, tissue doppler imaging, heart failure, coronary artery disease, 3D-TDI, triplane TDI, tissue-MPI.

## Abstract

Recent studies have explored the prognostic role of TDI-derived parameters in major cardiac diseases, such as coronary artery disease (CAD) and heart failure (HF). In these conditions, myocardial mitral annular systolic (S’) and early diastolic (E’) velocities have been shown to predict mortality or cardiovascular events. In heart failure non invasive assessment of LV diastolic pressure by transmitral to mitral annular early diastolic velocity ratio (E/E’) is a strong prognosticator, especially when E/E’ is > or =15. Moreover, other parameters derived by TDI, as cardiac time intervals and Myocardial Performance Index, might play a role in the prognostic stratification in CAD and HF. Recently, a three-dimensional (3-D) TDI imaging modality, triplane TDI, has become available, and this allows calculation of 3-Dvolumes and LV ejection fraction. We present a brief update of TDI.

## THREE MODALITIES: PW, COLOR MODE AND 3D-TDI

Tissue Doppler imaging (TDI) is an echocardiographic technique that uses Doppler principles to measure myocardial motion velocity.

Doppler echocardiography relies on detection of the shift in frequency of ultrasound signals reflected from moving objects. With this principle, conventional Doppler techniques assess the velocity of blood flow by measuring high-frequency, low amplitude signals from small, fast-moving blood cells. In TDI the same Doppler principles are used to quantify the higher amplitude, lower-velocity signals of myocardial tissue motion [1].

TDI can be performed in three modalities: pulsed wave, color mode and 3D mode.

Pulsed-wave TDI is used to measure peak myocardial velocities and is particularly well suited to the measurement of long-axis ventricular motion due to the longitudinally oriented endocardial fibers, which are more parallel to the ultrasound beam in the apical views. Since the apex remains relatively stationary throughout the cardiac cycle, mitral annular motion is a good surrogate measure of overall longitudinal left ventricular (LV) contraction and relaxation [2]. To measure longitudinal myocardial velocities, the sample volume is placed in the ventricular myocardium immediately adjacent to the mitral annulus. Pulsed-wave TDI has high temporal resolution but does not permit simultaneous analysis of multiple myocardial segments. 

With color TDI, a color-coded representation of myocardial velocities is superimposed on gray-scale 2-dimensional or M-mode images to indicate the direction and velocity of myocardial motion. Color TDI mode has the advantage of increased spatial resolution and the ability to evaluate multiple structures and segments in a single view [3].

Recently, a three-dimensional (3-D) TDI imaging modality, triplane TDI, became available, which allows calculation of 3-Dvolumes and LV ejection fraction. 

With 3D-TDI, a color-coded TDI is applied to the triplane view, obtained from the apical window in triplane modus, acquiring simultaneously the apical four-, two- and three-chamber views. At least two consecutive beats are recorded from each view, and the images are digitally stored for off-line analysis. 

To assess 3D volumes, the triplane dataset is frozen in end-diastole and the endocardial border is manually traced in the apical four-, two- and three-chamber, views respectively. Then, using the same heartbeat, the triplane dataset is frozen in end-systole and again the endocardial border is manually traced in the apical four-, two- and three-chamber views. A 3D LV end-diastolic and end-systolic volume is generated automatically by the software and LV volumes and ejection fraction are reported accordingly.

The triplane technique also permits to assess LV dyssynchrony, through the simultaneous acquisition of TDI from all LV segments during the same heartbeat. In this case, during post-processing analysis, the triplane TDI dataset is used to analyze myocardial velocity curves and then to calculate the time from the beginning of the QRS complex to peak systolic velocity (Ts), using the same heart beat. Subsequently, the standard deviation of Ts (Ts-SD) of the 12 myocardial segments is calculated and used as a parameter of LV dyssynchrony [4].

## TDI PARAMETERS

The PW Tissue Doppler Imaging allows to obtain several indices, which have clinical and prognostic implications. 

The cardiac cycle is represented by *three waveforms*:

1. Sa: systolic myocardial velocity above the baseline as the annulus descends toward the apex;
2. Ea: early diastolic myocardial relaxation velocity below the baseline as the annulus ascends away from the apex;
3. Aa: myocardial velocity associated with atrial contraction.

The lower-case “a” for annulus or “m” for myocardial (Ea or Em) and the superscripted prime symbol (E’) are used to differentiate tissue Doppler velocities from conventional mitral inflow. 

Systolic myocardial velocity (Sa) at the lateral mitral annulus is a measure of longitudinal systolic function and is correlated with measurements of LV ejection fraction and peak *dP*/*dt*.

Traditional echocardiographic assessment of LV diastolic function relied on Doppler patterns of mitral inflow. Because mitral inflow patterns are highly sensitive to preload and can change dramatically as diastolic dysfunction progresses, the use of mitral valve inflow patterns to assess diastolic function remains limited. TDI assessment of diastolic function is less load dependent than that provided by standard Doppler techniques. Validated against invasive hemodynamic measures, TDI can be correlated with τ, the time constant of isovolumic relaxation. In adults > 30 years old, a lateral Ea velocity> 12 cm/s is associated with normal LV diastolic function. Reductions in lateral Ea velocity to ≤ 8 cm/s in middle-aged to older adults indicate impaired LV relaxation and can assist in differentiating a normal from a pseudonormal mitral inflow pattern. Unlike conventional mitral inflow patterns, Ea is resistant to changes in filling pressure, although preload dependence is more pronounced in structurally normal hearts.

Simultaneous cardiac catheterization and echocardiographic studies have shown that LV filling pressures are correlated with the ratio of the mitral inflow E wave to the tissue Doppler Ea wave (E/Ea). The E/Ea ratio can be used to estimate LV filling pressures as follows: E/lateral Ea > 10 or E/septal Ea > 15 is correlated with an elevated LV end-diastolic pressure, and E/Ea < 8 is correlated with a normal LV end diastolicpressure.^3^

Although most studies refer to TDI’s focus on the measurement of velocities, some investigators have used TDI to obtain systolic and diastolic time intervals. TDI *time intervals*, measured from the sites (lateral or medial) at mitral annulus, are:

1. IVCT - isovolumic contraction time (from the end of the wave A’ to the beginning of the wave S’),
2. ET - ejection time (from the beginning to the end of the wave S’),
3. IVRT - isovolumic relaxation time (from the end of the wave S’ to the beginning of the wave E’),
4. St – systolic time (from the end of the wave A’ to the end of the wave S’),
5. Dt – diastolic time (from the end of the wave S’ to the end of the wave A’) [5].

Combining systolic and diastolic time intervals, we obtain the Myocardial Performance Index (MPI), defined as the sum of isovolumic contraction (IVCT) and relaxation time (IVRT) divided by the ejection time (ET).

This index, derived by conventional Doppler or Tissue Doppler, was reported to be simple and reproducible. One important limitation of conventional MPI is that the IVCT, IVRT and ejection time are measured sequentially and not on the same cycle. Consequently, the accuracy of the results may be compromised by heart rate fluctuations. Tissue Doppler echocardiography enables us to simultaneously measure both the diastolic and systolic intervals from the myocardium [6].

A possible disadvantage of conventional MPI is the effect of loading conditions on conventional MPI. Tei *et al* [7]. found a high correlation between conventional MPI and peak *dp/dt*, suggesting a relationship between MPI and preload. Thus, even when contractility is constant, significant changes in preload may cause significant alterations in conventional MPI. 

TDI, conversely, is relatively independent from the volume loading condition [8] and enables us to assess subclinical long-axis myocardial dysfunction that cannot be detected by conventional left ventricular systolic function measurements. 

Several studies have demonstrated that the Tei index obtained by TDI correlates well with the Tei index determined by pulsed Doppler [9].

The agreement between the methods in healthy subjects and in patients with dilated cardiomyopathy is high, while is slightly lower in patients with previous myocardial infarction (MI). Other studies have shown that the agreement between TDI and conventional method is not good, because systolic intervals are longer and diastolic intervals are shorter when measured with TDI. The disagreement exists on healthy people and it is increased on patients with prior myocardial infarction [10].

A study of healthy adults and patients with congestive heart failure has investigated the clinical agreement between MPI measured conventionally and by PW-TD of the mitral annulus. The results confirmed mild agreement between MPI measured by the conventional method and by PW-TDI. Both methods had similarly high diagnostic accuracy for cardiac heart failure; however, this study supports the use of a higher MPI cut-off point for best diagnostic accuracy when using the new PW-TDI method [11].

PW-TDI has also proved useful in the evaluation of the *isovolumic indices*. In the normal ventricle, there is segmental shortening before onset of left ventricular ejection, which contributes to changes in global left ventricular geometry and may mobilize blood that allows the mitral leaflets to bulge into the left atrium during systole. This pre-ejection shortening represents active contraction and most likely contributes to isovolumic velocities as measured by TDI. During isovolumic contraction (IVC) phase, tissue Doppler imaging studies suggest dynamic myocardial motions with biphasic longitudinal tissue velocities in left ventricular long-axis views. 

We can assess two indices: IVV - peak myocardial velocity during isovolumic contraction (cm/s) and IVA - myocardial acceleration during isovolumic contraction (m/s^2^). Peak IVA by TDI is calculated in different ways:

1. as the ratio of IVV divided by the acceleration time (AT, expressed in msec and calculated as the time from the beginning to the peak of the isovolumic wave occurring during the isovolumic contraction period) [12].
2. as the difference between the 2sequential IVC velocities, with the largest velocity increment divided by the frame-by-frame time interval [13].
3. from the derivative of the velocity curve (then allows detection of the pre-excitation region of the Wolff-Parkinson-White syndrome or other abnormalities in conduction).

Hashimoto *et al*. used a computerized method to measure acceleration and this approach might improve the accuracy of such measurements [14].

A study has assessed that peak positive myocardial velocity (IVV) of the left ventricular free walls, measured by PW-TDI during the IVC phase, correlates well with left ventricular global contractility. These observations have the potential to be of clinical value since disturbances of myocardial motion occur predominantly during the isovolumic phases, (i.e. during contraction and/or relaxation), and such non-invasive recordings might therefore be a sensitive marker of myocardial dysfunction [15]. In an animal study, Vogel *et al*. showed that IVC acceleration (IVA) is a reliable measurement of right ventricular contractility and is relatively load independent [16]. These authors concluded that IVC acceleration was a more sensitive marker of contractility than IVC peak positive velocity. In another study, Vogel *et al* have assessed the usefulness of IVA to measure left ventricular contractile function and force-frequency relationships in an experimental preparation. They have demonstrated that IVA is a measurement of left ventricular contractile function; furthermore it is unaffected by preload and afterload changes within a physiological range and can be used noninvasively to measure left ventricular force-frequency relationships [17].

Then IVV (isovolumic velocity) and IVA (isovolumic acceleration) are an additional non-invasive tool to be taken into consideration for complete assessment of left ventricular contractility. However, the usefulness of IVV and IVA in the early detection of myocardial dysfunction needs to be clarified [18].

The feasibility, reproducibility and variation of IVA between segments have not been studied in detail, and thus its utility in clinical practice has not been established.

Recently, a study has confirmed that IVA may be used as a research tool if it is measured at the medial mitral annulus, but its clinical applicability is hampered by low reproducibility, especially in patients with impaired left ventricular function in whom it would otherwise be most useful [19].

TDI is also used to assess global left ventricular *mechanical dyssynchrony *and to determine the response in left ventricular structure and function after cardiac resynchronization therapy. An index of dyssynchrony, originally described by Yu and co-workers [20, 21], is the standard deviation of the time to peak myocardial systolic contraction based on a 12-segment model of LV (Ts-SD). Time to peak is assessed analyzing the time from the beginning of QRS to the peak of systolic wave (Sa).

In our experience, we found that there is a learning curve associated with the understanding and utilization of TD imaging. Differences in obtaining and interpreting TD data were observed between experienced and non-experienced TD operators. All operators could in fact easily evaluate systolic velocity (S’), early (E’) and late (A’) diastolic velocities, the ratio of early to late diastolic velocity (E’/A’) and the transmitral to mitral annular early diastolic velocity ratio (E/E’), but the different time intervals calculated by Tissue Imaging (Isovolumic Contraction Time, Ejection Time, Isovolumic Relaxation Time) and tissue Myocardial Performance Indexwere achieved only by experts in TD echocardiography [22].

### Coronary Artery Disease

TDI has been introduced as a method to quantify myocardial function in terms of tissue velocities and the results so far are promising.

As demonstrated in animal models and in patients with coronary artery disease, myocardial ischemia is characterized by a decrease in peak systolic myocardial velocity (S’), indicating impairment of regional contractile function. There are important limitations in the ability of peak systolic velocity to serve as a quantitative marker of regional function. Furthermore, a more comprehensive analysis of the myocardial Doppler velocity signal may improve the ability of TDI to identify ischemic myocardium. This analysis includes measurement of *myocardial velocities during isovolumic contraction phase and isovolumic relaxation phase* in addition to ejection velocities. 

In the *non ischemic ventricle* the isovolumic contraction period (IVCT) is dominated by a positive velocity spike of short duration, which represented slight longitudinal shortening before LV ejection. During isovolumic relaxation time (IVRT), there is a pattern opposite to that during isovolumic contraction period, with a negative velocity spike of short duration, representing slight elongation before onset of filling. Possibly, twist in gand untwisting effects may have contributed to the IVC and IVR velocities.

#### During moderate ischemia,

there is a decrease in systolic shortening and then a decrease in peak early ejection and mid-ejection velocities.

#### During severe ischemia,

early-ejection Doppler velocities remain positive and therefore don’t reflect the marked impairment of myocardial function. The mechanism of the positive early-ejection velocity in dyskinetic myocardium is not clear, but it might represent cardiac translational motion or tethering effects resulting from contractions in other myocardial segments.

The dominant systolic velocity component during *severe ischemia *is a large negative velocity spike during isovolumic contraction period. During severe ischemia, IVR velocities reverse and a large positive velocity component (post systolic shortening of ischemic myocardium) persists throughout the entire IVR period, and in some cases continues after the early-diastolic LA/LV pressure crossover.

Then, in severely ischemicand dyskinetic myocardium, IVC and IVR velocities arethe strongest markers of myocardial dysfunction. Myocardial ejection velocities have very low amplitudes and appear to be influenced by tethering effects and/or translational motion [23].

Some studies have investigated the useful of the *time intervals* in the assessment of coronary artery disease.

In patients with acute myocardial infarction the *conventional Tei index* was found to be significantly more greater than in healthy controls [24].

A study, in patients with prior myocardial infarction, has assessed the degree of agreement between PWD and a method based on tissue Doppler imaging. TDI intervals may be influenced by intraventricular conduction disturbs, asynchrony and the differences in the contraction and relaxation times between the different myocardial segments. Mitral annulus velocity intervals, in particular, could be highly influenced by the contractility of basal segments. In these patients, the differences between conventional MPI and TDI-MPI were similar in patients with good contractility of basal segments and even in patients without segmentary contractility alterations. Furthermore, similar observations could be found in healthy subjects. Therefore the disagreement between methods does not seem to depend on contractility alterations. The values of IVCT and ET intervals measured with TDI were larger than the ones obtained with conventional Doppler; this could increase TDI-MPI values. On the other hand, IVRT interval was usually shorter with TDI, and this could decrease TDI-MPI values. The results of both the influences were values of MPI and TDI-MPI of similar range, but not accurate ones. Of the intervals needed to calculate TDI-MPI, ET showed lower differences with the conventional method, while IVCT and IVRT correlated poorly with classic intervals. Therefore, isovolumic times are in most part the responsible components of MPI which explain the differences between conventional MPI and TDI-MPI [10].

TDI-MPI has proved useful in the assessment of left ventricular thrombosis risk after acute myocardial infarction (AMI). Particularly when an MPI > 0.6 was used as the cutoff, LV thrombus formation could be predicted with a sensitivity rate of 81%, a specificity rate of 73%, a positive predictive value of 62%, and a negative predictive value of 88% [25].

Baykan *et al* have evaluated the systolic and diastolic function of left ventricle by PW-TDI in patients with or without preinfarction angina in acute myocardial infarction. The patients with preinfaction angina showed values of E' and E'/A' higher than patients without preinfarction angina, while the ratio E/E' and the MPI were significantly lower in the first group of patients. So the diastolic function was better in patients with preinfarction angina than patients without [26].

### Heart Failure

A variety of indices derived from Doppler-echocardiography have been used to predict outcome in patients with heart failure. Recently, tissue Doppler imaging (TDI) has been used to assess systolic and diastolic function [27]. Several studies have shown that the early mitral annulus velocity is a relatively preload-independent assessment of LV relaxation [28, 29] and the ratio of peak early diastolic mitral inflow velocity (E) over the myocardial velocity can be used to estimate LV filling pressure [19-31]. 

Wang *et al*. examined whether TDI derived parameters added incremental value to clinical and other standard Doppler-echocardiographic measurements to predict cardiac mortality in patients with a variety of cardiac diseases and ventricular function. They showed that the TDI derived parameters Sm, Em, and Am are powerful predictors of cardiac mortality. In particular an Em<3 cm/s, Sm<3 cm/s, Am <4 cm/s, and E/Em>20 can identify patients at very high risk of cardiac death in the subsequent two years [32].

Yip *et al* have tested the hypothesis that, when measured in the long axis, left ventricular systolic function is abnormal in patients with diastolic heart failure. The mitral annular peak mean velocity and amplitude in systole were lower in the patients with diastolic heart failure (mean (SEM), 4.8 (0.2) cm/s) than in the age matched normal controls (6.1 (0.14) cm/s), but higher than those with systolic heart failure (2.8 (0.13) cm/s) (all p < 0.001). Similar changes were seen the mitral annular amplitude during systole. Peak early diastolic velocity and amplitude were also significantly reduced in the group with diastolic heart failure. This study has showed that in patients with diastolic heart failure and evidence of left ventricular hypertrophy, there is systolic left ventricular impairment as measured by myocardial Doppler imaging of the longitudinal axis. Thus subtle abnormalities of systolic function are present in patients with heart failure and a normal left ventricular ejection fraction, and there appears to be a continuum of systolic function between those with truly normal, mildly impaired (labelled diastolic heart failure), and obviously abnormal left ventricular systolic function [33].

Recently, the ratio of transmitral E velocity to early diastolic mitral annular velocity (E/E') has been shown to be useful to assess LV filling pressure. A persistent elevation of E/E’ ratio may be an indicator of patients with or at risk of DHF among subjects with preserved systolic function independent of LV hypertrophy [34].

In diastolic heart failure patients persistent E/e’>15 is an index of higher clinical events. Then elevated E/E' after optimized medical therapy may be useful in predicting cardiac events in patients with HFPSF (heart failure with preserved systolic function) [35].

Hills *et al* have hypothesized that an E/e' ratio >15 would predict poorer survival after acute MI. An E/e' >15 was associated with excess mortality and was the most powerful independent predictor of survival (risk ratio 4.8, 95% confidence interval 2.1 to 10.8, p = 0.0002). The addition of E/e' >15 improved the prognostic utility of a model containing clinical variables and conventional echocardiographic indexes of left ventricular systolic and diastolic function (p = 0.001). Noninvasive estimation of left ventricular filling pressure by E/e' is a powerful predictor of survival after acute myocardial infarction [36].

TDI *time intervals* and Myocardial Performance Index might play an important role in the diagnosis and prognosis of heart failure. 

In patients with dilated cardiomyopathy, the *conventional MPI* is found to reflect the severity of LV dysfunction and was proved to be an independent prognostic factor for mortality, similar to the EF. The higher Tei index values in these patients than in healthy individuals are attributable to prolongation of the isovolumic intervals and shortening of ET. The Tei index is significantly correlated with NYHA class, EF and ventricular volumes, while values > 0.77 are associated with higher 1-, 3- and 5-year mortality [37]. The usefulness of the index was studied in the detection of patients with mild to moderate heart failure. The Tei index was significantly greater in patients with heart failure than in controls and was correlated with LV end-diastolic pressures. Values > 0.47 identified heart failure patients with a sensitivity of 86% and a specificity of 82% [38]. Harjai *et al*. investigated the prognostic value of the Tei index in 60 patients with severe, symptomatic heart failure (EF < 30%) of ischemic aetiology or not. A Tei index >1.4 was an independent prognostic factor for death or emergency heart transplant during two years’ follow up and had more predictive power than EF or NYHA class [39].

Tissue Doppler echocardiography enables us to evaluate new parameters and allows greater prognostic stratification of patients with heart failure.

The length of time intervals, derived from TDI, varies by type of heart failure, systolic or diastolic. In fact, the duration of the systolic contraction time was statistically shorter in diastolic heart failure patients compared with controls and still shorter in the systolic heart failure group.

Instead, the isovolumic relaxation time (IVRT) and isovolumic contraction time (IVCT) tend to increase in diastolic heart failure and even more in the systolic. Consequently, the MPI will tend to increase in diastolic and systolic heart failure [40].

In a study conducted on a population of 112 patients (II-III NYHA class, sinus rhythm, systolic dysfunction identified by FE≤45%) value of MPI ≥ 0.55 was associated with a relative risk of adverse cardiovascular events of 18,7 [41].

In another study, conducted on a population of patients with predominant diastolic heart failure, an MPI > 0.59 might be useful in identifying subjects with concomitant systolic impairment and neurohormonal activation [42].

Therefore the MPI correlates with the occurrence of adverse events in the follow up of patients with chronic heart failure. In fact, a poor outcome in subjects with heart failure and with an MPI > 0.90 was demonstrated [43].

In our study we found that TDI time intervals may be able in stratifying the risk of recurrent hospitalization for acute HF in subjects with chronic HF. Re-hospitalizations for acute HF were predicted by lower values of ET, ST and FT, and higher values of MPI, and ICT/ET. ET and ST values ability to predict future adverse events remained significant even after correction for LVEF and several other traditional prognostic factors in chronic HF; their assessment in subjects with preserved LVEF may be therefore useful in identifying patients with a higher risk of hospitalization, regardless of LVEF. In particular, ET ≤236 ms identified subjects with occurrence of re-hospitalization for acute HF with a specificity of 78% and a negative predictive power of 93%, FT≤317 ms with a specificity of 69% and a negative predictive power of 94%, ST≤306 ms with a specificity of 90% and a negative predictive power of 91%, ICT/ET values below 0.72 with a specificity of 94% and a negative predictive power of 91% [44].

Exact mechanisms by which TDI intervals are related with prognosis in chronic HF are not completely known. Recently we showed as, in ischemic chronic HF patients, therapy with statins is related with a better TDI performance (higher values of ET and ST) and prognosis ^i^. These data might therefore suggest possible mechanisms by which statins exert part of their positive action on dysfunctional ventricles of patients with chronic HF [45].

Few studies have evaluated the useful of *isovolumic indices* in heart failure. Recently was explored the incremental value of quantification of tissue Doppler velocity during the brief isovolumic contraction phase of the cardiac cycle for the prediction of exercise performance in patients with reduced left ventricular ejection fraction (LVEF), referred for cardiopulmonary exercise testing (CPET). Patients with reduced EF had lower IVV, Sa and Aa velocities. Similarly, % predicted peak VO_2_was lower in patients with reduced EF and correlated with the variations in IVV. Then assessment of TD-derived IVC and atrial stretch velocities provide independent prediction of exercise capacity in patients with reduced LVEF. Assessment of LV pre-ejectional stretch and shortening mechanics at rest may be useful for determining the myocardial functional reserve of patients with reduced EF [46].

Despite recent advances in management, chronic heart failure (CHF) is still associated with a high morbidity and mortality. Cardiac resynchronization therapy (CRT) has been established as an adjunctive treatment for patients with left ventricular systolic dysfunction and medically refractory heart failure with a wide QRS interval. The CRT, reducing ventricular asynchrony, improves cardiac performance, reduces mitral regurgitation, reduced filling pressures and induces reverse remodeling of the left ventricle.

However up to 40% of patients who receive a CRT device for established indications do not respond to CRT. In the search for better selection criteria for CRT, it has been shown that patients with LV dyssynchrony have a higher likelihood of a positive response to CRT [6].

The most frequently used technique is TDI, which can be used to determine the response in left ventricular structure and function after device implantation and emerging evidence as a method for selection of patients who may derive clinical benefit from CRT. Several indices of LV dyssynchrony have been proposed as predictors of response to CRT, but the recently published PROSPECT trial failed to identify an ideal echocardiographic parameter of dyssynchrony [47].

The assessment of intraventricular dyssynchrony can be done through various indices, including one proposed by Yu and co-workers [20, 21]. This index is the standard deviation of the time to peak myocardial systolic contraction based on a 12-segment model of LV (Ts-SD). A larger value of SD correlates with more severe left ventricular (LV) asynchrony in patients with heart failure.

To calculate this parameter of LV dyssynchrony, three apical views need to be acquired separately. A 3-dimensional (3D) TDI imaging modality (tri-plane TDI) has become available to overcome this limitation. Tri-plane TDI allows simultaneous acquisition of all LV segments during the same heartbeat rendering the technique more precise than the 2-dimensional (2D) TDI equivalent. Then a 3-D probe has become available and useful to identify responders to cardiac resynchronisation therapy (CRT).

A study has evaluated the value of triplane TDI to predict reverse left ventricular (LV) remodelling after CRT. Sixty patients with heart failure, scheduled for CRT, underwent triplane echocardiography with simultaneous TDI acquisition before and 6 months after implantation. Intraventricular dyssynchrony was quantitatively analysed by evaluating time from onset of the QRS complex to peak myocardial systolic velocity in 12 LV segments from the triplane dataset and calculation of the standard deviation (Ts-SD-12). Clinical response was defined as an improvement of at least one New York Heart Association class. Reverse LV remodelling was defined as ≥ 15% decrease of LV end-systolic volume at 6 months' follow-up. This study has demonstrated that triplane TDI echocardiography predicts clinical response and reverse LV remodelling 6 months after CRT implantation [4].

As stated above, to obtain myocardial velocity curves for 12 LV segments with 2D-TDI, multiple acquisitions are essentially required. Thus, TDI analysis of dyssynchrony has been applicable only to patients with sinus rhythm fitting into the setting of regular RR intervals for three apical images. Assessment of SD in patients with atrial fibrillation (AF), characterized by randomly irregular cycle lengths, theoretically requires simultaneous image acquisition in three planes. Although AF is associated in large portions of patients with heart failure, many studies evaluating LV asynchrony or response to the cardiac resynchronization therapy (CRT) have excluded patients with AF because of the lack of standard method by conventional echocardiography. In this regard a study has demonstrated the feasibility of the new triplane TDI method for measuring the SD in patients with AF. The authors have proved that the eight-cycle average SD for 12 LV segments showed significant correlation with echocardiographic parameters of systolic function. This method should be further validated, and the mechanism and clinical impact of dyssynchrony in patients with AF should be investigated in further studies [48].

Information on cardiac dyssynchrony can also be derived from *nuclear imaging*. In particular, a recent development is the use of gated myocardial perfusion single photon emission computed tomography (GMPS) for assessment of LV dyssynchrony. Recently, Chen *et al*. [49] developed with GMPS a count-based method to extract phase information from the regional LV count changes throughout the cardiac cycle. The authors assessed in 90 normal individuals the normal range for *four quantitative indices* that can be used as markers of LV dyssynchrony(histogram bandwidth, phase SD, histogram skewness and histogram kurtosis). Recently, these four GMPS indices have been compared with LV dyssynchrony assessment by TDI in 75 patients with severe heart failure. It was shown that among the four quantitative indices of phase analysis, the variables histogram bandwidth and phase SD correlated best with LV dyssynchrony as assessed by TDI [50]. The current study involved a different patient subset and provides further support that phase analysis with GMPS can be useful in the evaluation of LV dyssynchrony.

To further validate the use of GMPS with phase analysis for the assessment of LV dyssynchrony, Marsan *et al*. performed a direct comparison with tri-plane TDI in a cohort of heart failure patients. The results of this study confirm the feasibility to evaluate LV dyssynchrony with phase analysis by GMPS and its applicability in the clinical setting. In particular, histogram bandwidth and phase SD showed a good correlation with LV dyssynchrony measured with Ts-SD as assessed with tri-plane TDI. Future prospective studies in larger patient populations with follow-up after CRT implantation are needed to elucidate the potential role of GMPS with phase analysis for prediction of response to CRT [51].

An other method to assess global left ventricular mechanical dyssynchronyis the real-time 3D echocardiography (RT3DE) [52].

A study has assessed the comparability of left ventricular mechanical dyssynchrony assessment by TDI and RT3DE in patients with a wide range of LV ejection fractions and different causes of cardiomyopathy. In addition, this study has evaluated the ability of both techniques to predict response to cardiac resynchronization therapy (CRT). Mechanical dyssynchrony was measured with TDI using the standard deviation of time to peak systolic tissue velocity of 12 LV myocardial segments. With RT3DE, the standard deviation of time from QRS onset to minimal volume of 16 LV subvolumes was assessed. The results have confirmed that marked differences between techniques are found for the presence of mechanical dyssynchrony when current cutoff values are applied, making interchangeability of these techniques uncertain. Furthermore assessment of mechanical dyssynchrony by RT3DE might be an appropriate alternative to TDI for accurate prediction of response to CRT [53].

Other parameters, useful to identify the responders to CRT, are the TDI time intervals. In a previous study, the baseline LV Tei index was significantly higher in responders and exhibited an acute and sustained improvement after CRT. The baseline RV Tei index was similar in responders and non responders but improved significantly only in responders [54].

The results of another study indicate that the Tei index and E/E’ ratio are independent predictors of poor response and cardiac events after CRT [55].

Recently, Porciani *et al* have evaluated the predictive value of echo/Doppler derived indices, which reflect the duration of the isovolumic phases of the cardiac cycle, in identifying CRT responders. In 105 patients, before and 6 months after CRT, the following echo/Doppler parameters were evaluated: myocardial performance index (MPI) and total isovolumic time (t-IVT) as the sum of IVCT and IVRT divided by the RR interval (obtained by mitral pulse wave Doppler); standard deviation of the time to systolic peak velocity (Ts-SD) (obtained by TDI) as asynchrony index. At baseline, responders had higher t-IVT and MPI than non responders. Receiving operating characteristic curve analysis showed that both t-IVT and MPI could predict CRT response. Then Echo/Doppler derived indices, describing physiologic abnormalities of the isovolumic contraction and relaxation phase, are able to predict CRT-induced reverse remodeling [56].

Rocchi *et al*. [57] have demonstrated that exercise intraventricular dyssynchrony, assessed during exercise TDI ECHO, is a strong predictor of CRT response, significantly superior to rest intraventricular dyssynchrony. In their work, it was the only parameter independently associated with LV reverse remodelling after CRT at multivariable analysis (*P*< 0.001) and was clearly superior to rest LV dyssynchrony in predicting functional improvement at 6 min walking test. Exercise TDI ECHO showed also an overall predictive value for CRT response of 92%. It could therefore be used to improve selection criteria for patients who are candidates for CRT, thus reducing the frequent inappropriate implantation of biventricular pacemakers. 

## CONCLUSION

PW-TDI is a useful complement to standard echo-Doppler examination and a helpful tool in the diagnosis and prognosis of CAD and HF. TDI-MPI is considered a reliable parameter for evaluation of global left ventricular function. PW-TDI has proved useful in the evaluation of the isovolumic indices, which correlate well with left ventricular global contractility. TDI is used to assess global left ventricular mechanical dyssynchrony and to determine the response in left ventricular structure and function after cardiac resynchronization therapy.

## DISCLOSURE

Part of information included in this article has been previously published in INTERNAL AND EMERGENCY MEDICINE Volume 6, Number 5, 393-402, DOI: 10.1007/ s11739-010-0469-3.

## Figures and Tables

**Fig. (1):**
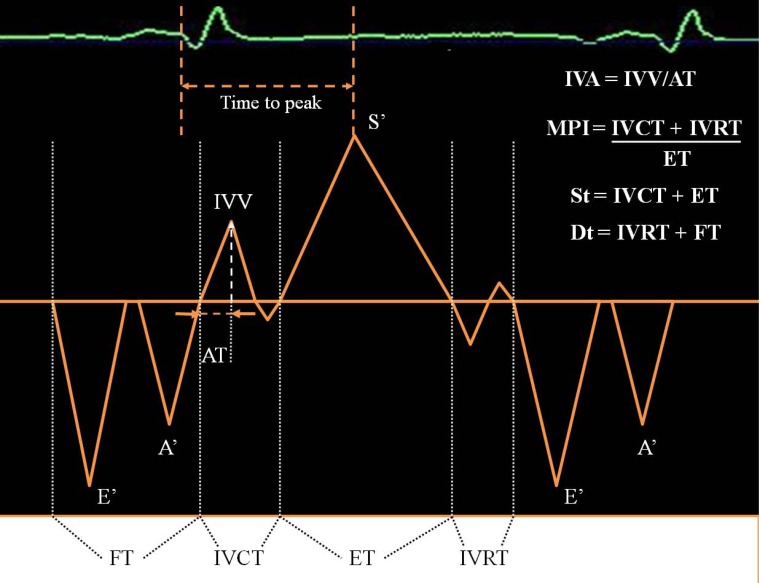
Diagram of TDI-derived waves and cardiac time intervals: **S’** – systolic wave, **E’**–earlydiastolic wave, **A’** - late diastolic wave, **IVV** - myocardial velocity during isovolumic contraction, **IVA** -myocardial acceleration during isovolumic contraction, **AT** – acceleration time, **FT** – filling time, **IVCT** - isovolumic contraction time, **ET** - ejection time, **IVRT** - isovolumic relaxation time, **St** – systolic time, **Dt**– diastolic time, **MPI** – myocardial performance index or Tei index.

**Fig. (2):**
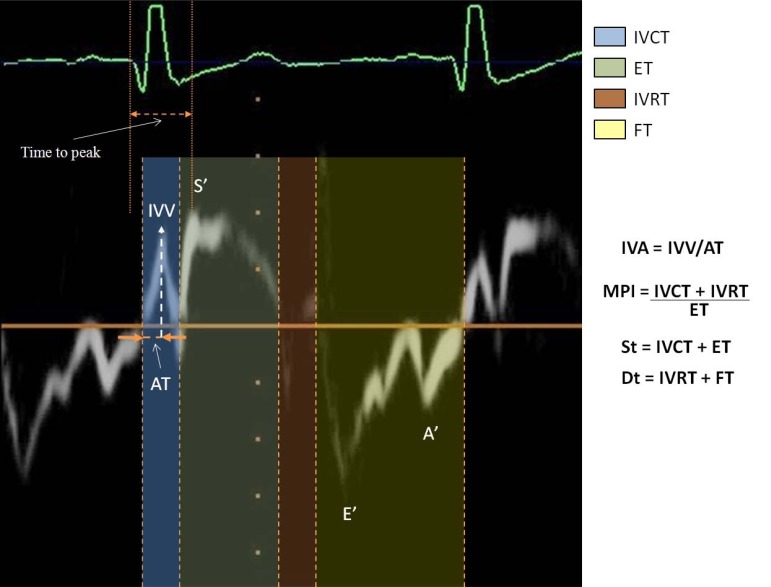
Spectrogram of PW-TDI. In particular: Tissue Doppler – derived myocardial systolic and diastolic velocities, and time intervals.

**Fig. (3):**
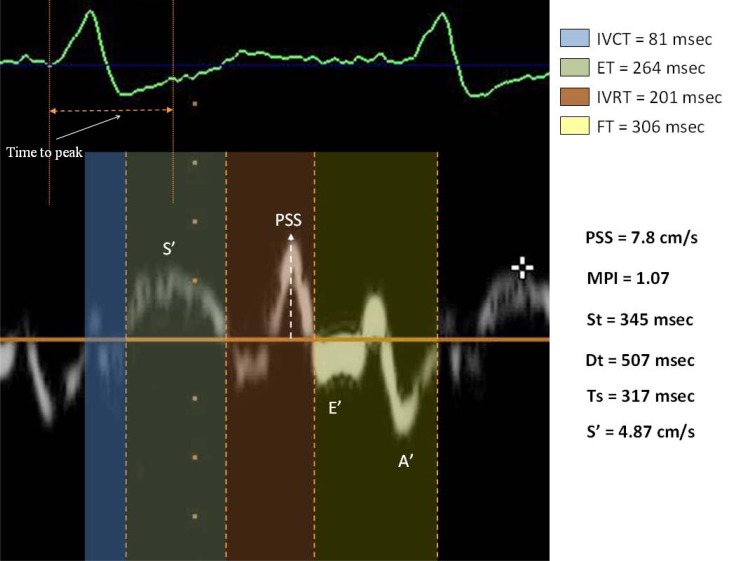
TDI parameters in patient with CAD. PSS: post systolic shortening.

**Fig. (4):**
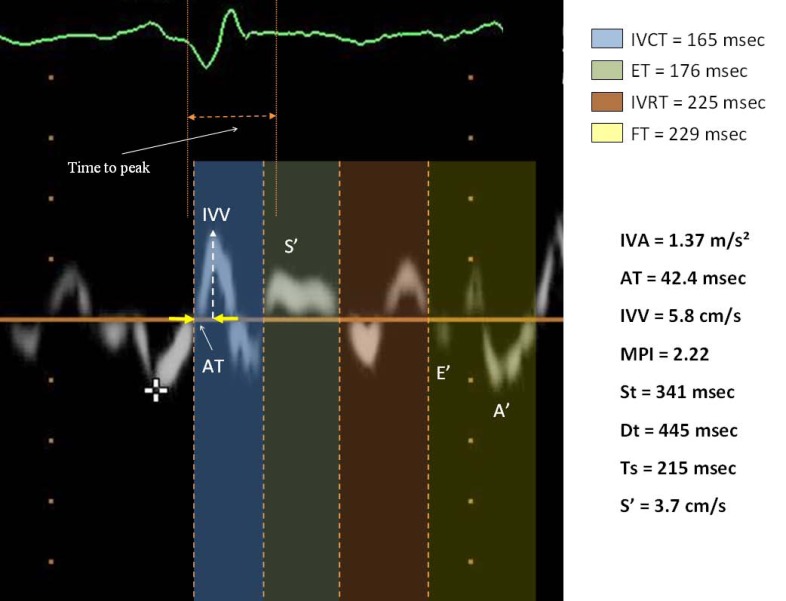
TDI parameters in patient with systolic HF.

## ABBREVIATIONS

TDI: = Tissue Doppler Imaging
MPI: = Myocardial Performance Index or TEI index
Sa (Sm or S’): = Systolic wave
Ea (Em or E’): = Early diastolic wave
Aa (Am or A’): = Late diastolic wave
IVCT: = Isovolumic contraction time
IVRT: = Isovolumic relaxation time
ET: = Ejection time
FT: = Filling time
St: = Systolic time
Dt: = Diastolic time
IVV: = Myocardial velocity during isovolumic contraction
IVA: = Myocardial acceleration during isovolumic contraction
Ts-SD: = Standard deviation of the time to peak myocardial systolic contraction
